# Biocuration: Distilling data into knowledge

**DOI:** 10.1371/journal.pbio.2002846

**Published:** 2018-04-16

**Authors:** International Society for Biocuration

## Abstract

Data, including information generated from them by processing and analysis, are an asset with measurable value. The assets that biological research funding produces are the data generated, the information derived from these data, and, ultimately, the discoveries and knowledge these lead to. From the time when Henry Oldenburg published the first scientific journal in 1665 (*Proceedings of the Royal Society*) to the founding of the United States National Library of Medicine in 1879 to the present, there has been a sustained drive to improve how researchers can record and discover what is known. Researchers’ experimental work builds upon years and (collectively) billions of dollars’ worth of earlier work. Today, researchers are generating data at ever-faster rates because of advances in instrumentation and technology, coupled with decreases in production costs. Unfortunately, the ability of researchers to manage and disseminate their results has not kept pace, so their work cannot achieve its maximal impact. Strides have recently been made, but more awareness is needed of the essential role that biological data resources, including biocuration, play in maintaining and linking this ever-growing flood of data and information. The aim of this paper is to describe the nature of data as an asset, the role biocurators play in increasing its value, and consistent, practical means to measure effectiveness that can guide planning and justify costs in biological research information resources’ development and management.

## Data as an asset

Research data continue to be produced at ever-growing rates due to both technological advances and decreasing costs for their generation [1]. Understanding what makes data assets distinct from other types of assets is fundamental in terms of their valuation and effective management [2]. To briefly summarise, from an economic perspective, its unique characteristics are these: Information is infinitely shareable without any decrease in its intrinsic value. For example, the same sequence retrieved from the National Center for Biotechnology Information (NCBI) can be shared by an unlimited number of people without any loss of value. Unlike physical assets—e.g., sequencing equipment, which depreciates with use—information sharing actually increases its value in a compound fashion; and reciprocally, unshared information is less valuable [3,4,5]. Further, the more accurate and complete the information is, the more valuable it is. In other words, quality is at least as important as quantity [6,7,8]. Since inferences are only as good as the information they are based upon, inaccuracies and omissions compel scientists to spend valuable research time winnowing out poor-quality or inaccurate information or, even worse, inadvertently ploughing research funds into dead ends. Moreover, with the increasing role of automatic inference systems for high-throughput data and data analytics, there is a growing dependency on the availability of robust, high-quality knowledge resources, and the gold-standard data sets they contain, for benchmarking. Lastly, when information is combined, its value increases. For example, genetic testing can reveal hundreds of thousands of variants per individual, yet for most variants, the clinical consequences are not yet known [9]. If our goal is to advance research, instantiation of known connections is essential to accelerate the process of relating genotypes to phenotypes in a way that is impossible when using individual data sets in isolation [10,11,12,13,14].

Managing a biological information resource relies on a range of intersecting skills: Bioinformaticians, application developers, system administrators, biocurators, journal editors, etc. are all involved in this collective effort. Within this context, biocurators focus on information content rather than technology. Their overarching goal is to maximise the value of the information assets researchers are generating by assuring their accuracy, comprehensiveness, integration, accessibility, and reuse.

## What is biocuration?

Biocuration is the extraction of knowledge from unstructured biological data into a structured, computable form. In this context, knowledge is most commonly extracted from published manuscripts, as well as from other sources such as experimental data sets and unpublished results from data analysis. Biocurators are typically PhD-level biologists, often with lab bench experience coupled with specialised expertise in computational knowledge representation. Their work entails the synthesis and integration of information from multiple sources—including, for example, peer-reviewed papers; large-scale projects, such as the Encyclopedia of DNA Elements (ENCODE); or conference abstracts. They contact authors directly for clarification, digest supplemental information, and resolve identifiers in order to accurately capture a researcher’s conclusion and their evidence for that conclusion. Biocurators strive to distil the current ‘best view’ from conflicting sources and ensure that their resources provide data that are not only findable, accessible, interoperable, and reproducible (FAIR), but also traceable, appropriately licensed, and interconnected (collectively, the FAIR-TLC principles [15]).

## Biocuration motivation

Scientific communication is shifting in this ‘information age’, with researchers increasingly relying on curated resources [16,17,18,19]. For example, when comparing an entry in the Worldwide Protein Data Bank (wwPDB; https://www.wwpdb.org)—a resource containing detailed reviewed information on macromolecular structures—with a portable document format (PDF) file containing a figure of the same structure, it is obvious that the latter, non-computer-readable representation is insufficient for downstream comparative use. The political processes in the scientific community that led to designating wwPDB [20], the International Nucleotide Sequence Database Collaboration [21], and others such as the International Molecular Exchange (IMEx) [22] and ProteomeXchange consortia [23] as official depositories have proven to be well worth the effort. These examples highlight the importance of collaboration and synergy between journal editors and databases. The definition of what it means to publish is expanding [24], since results only published as a PDF have limited accessibility. To promote impact and reuse, the full semantic spectrum must be employed, from human-readable language to fully computationally interpretable.

## Biocuration costs

Although expert biocuration is clearly labour intensive, it scales surprisingly well with the growth of biomedical literature, as demonstrated by two recent studies [25,26]. Advanced tools are also increasing efficiency and accuracy, and biocurators are often actively engaged as team members in developing machine learning and natural-language processing techniques. Although these methods currently lack the necessary precision and recall required for a real-world setting [27,28,29], they are beginning to provide assistance [30,31,32,33,34] and will continue to incrementally improve.

The costs for sustaining a useful research resource in which biocuration plays an essential role represent only a tiny fraction of the original research funding [35]. An independent survey assessing the value of biological database services concluded that the benefits to users and their funders are equivalent to more than 20 times the direct operational cost of the institute [36]. Additionally, the hidden cost of an individual researcher’s time spent trawling the literature to find the information pertinent to their own specialist field is impossible to estimate, but having the required data easily accessible in a structured format represents a considerable saving in person-hours and, therefore, money for every funder, academic institute, and biomedical enterprise.

## Actionable recommendations

### Everyone can be a biocurator—Data reporting fit for knowledge synthesis

Seriously addressing seemingly mundane issues—such as identifying gene symbols, isoforms, strains, antibodies, and cell lines—is essential if experimental results are to be correctly integrated within the existing body of knowledge. For example, a recent study found that almost 40% of the gene lists submitted to the Gene Expression Omnibus (GEO) and 20% of the gene lists in the supplementary material of published articles contain gene symbol errors introduced by the software used during data handling prior to publication [37]. This will continue to be a significant problem until infrastructure is in place at key junctions in the research life cycle. New tools and workflows are needed for connecting researchers, journals, reviewers, and repositories and easily conveying standards-compliant information.

Progress is being made; notably, community guides for provisioning and referencing life science identifiers have recently been published [38,39], outlining best practices for facilitating large-scale data integration. Likewise, in the lab, software applications that support autocompletion within individual cells of spreadsheets, as well as more sophisticated standards-aware data collection tools, ensure that standard terminologies are applied as data are collected [40,41,42]. Through the use of such electronic laboratory notebook and manuscript submission software and the adoption of recommended formats and community-endorsed terminologies and ontologies, the goal of ‘born computable’ lab data generation will be realised. Initiatives have also started in scientific journals. A good example is provided by SourceData, a project initiated by the European Molecular Biology Organization (EMBO) press, which involves the biocuration of article figures prior to publication [43].

### Support for standards—Development, usage, and sustainability

Common standards for describing and classifying biology are indispensable for reproducible interactions, information exchange, interoperability, comparability, and discoverability [44]. Without standards, database search results will inevitably miss key information or include irrelevant material.

Biocurators regularly lead efforts in standards development: engaging with experts, building consensus, fostering adoption, and maintaining biological fidelity. Yet apart from a very limited number of cases, funding for standards development is unavailable. Even in the case of the Gene Ontology Consortium [45], the funding for this indispensable standard is significantly aided through other projects. On the other side of the spectrum, the Human Phenotype Ontology [46,47,48] operates using donated time from a handful of dedicated individuals, despite its widespread adoption (e.g., the Unified Medical Language System [UMLS], United Kingdom 100,000 Genomes Project, and the Global Alliance for Genomics and Health [GA4GH]). While the lack of dedicated funding poses a risk, the harmful consequences of not using any standard are vastly greater.

More can be done to inform and educate data producers and consumers on the importance of standards to ensure research data are not wasted or lost in the wrong format, with the wrong metadata descriptions, or described using a private or personal set of terms. Efforts such as FAIRsharing [30] (fairsharing.org), which maps the landscape of databases and standards and links them to the journal and funder data policies that endorse their use, go a long way to making sure that existing standards are adopted. However, more funding is needed for these infrastructure projects to aid data and knowledge sharing, to minimise the duplication of effort, and to ensure that researchers can easily employ appropriate standards.

### Expediting the collection and processing of data

Recently, there has been considerable excitement about the strategy of crowdsourcing, putting biocuration tools into a researcher’s hands so that they may directly contribute and publish their results into knowledge resources [49,50,51,52]. There is a tremendous potential in this approach, but to ensure success, there are clear prerequisites that must be satisfied—(i) editorial oversight, (ii) automated integrity checks, and (iii) citation mechanisms. Successful community-sourced projects universally include editorial control, which is where biocurators can play a key role, to avoid collecting poor-quality data that would decrease the value of a resource overall.

In addition, support for developing user interfaces, batch submission tools, and utilities to computationally validate content—such as simple checks for syntactical correctness, falling outside standard deviations, or using disallowed values—is needed for direct data submission. Here again, biocurators often play a role in defining validation standards. Machine-readable standards are critical in this step, as they enable validation to be carried out programmatically. Continuous integration and contextual analysis approaches may even suggest what a contributor might do to improve their data before making a final submission. Notably, biologists are already beginning to use community curation tools when they are available, such as Canto [53]—which is used by researchers working on *Schizosaccharomyces pombe* to directly submit their data to a resource—and Apollo [54], which is used for community-based curation of gene structures for improving automated gene sets.

Lastly, citation mechanisms need to be built into the contribution process. This both acts as an incentive and fosters reproducibility, since information is traceable to the original experimental work that led to a conclusion. Currently, existing biological data resources associate every assertion they contain with its underlying experimental justification by linking it to a PubMed identifier, which is an indirect route to the actual researcher(s) who contributed this information. Literature citations are mere proxies for assessing productivity and impact. Embedding a traceable authorship facility directly into laboratory software or a resource’s submission software would provide a much more direct and accurate means of assessing a researcher’s impact. By associating a researcher (e.g., an Open Researcher and Contributor ID [ORCID] persistent identifier, https://orcid.org/) with an identified piece of information (e.g., a persistent identifier, such as a digital object identifier [DOI]), their contributions become citable objects [55,56,57], and the subsequent use of this information by other researchers can be tracked. If this is encouraged, one can envision a time when community curation tools become the first place for digitally publishing research conclusions, shared directly into digital community resources.

### Biocuration is a necessity for scientific progress

Actively promoting innovations in fundamental data and information capture will yield enormous return on our research investment. The existing pain points—the time wasted by individual researchers discovering information, collecting it, manually verifying it, and integrating it in a piecemeal fashion—all impede scientific advancement. For researchers, biocuration means they can easily find extensive and interlinked information at well-documented, stable resources. It means they can access this information through multiple channels by browsing websites, downloading it from repositories, or retrieving it dynamically via web services. It likewise means the information will be as accurate and reliable as possible. And—because biocurators have integrated information by describing it using community semantic standards, applying authoritative identifiers, and transforming it into standard formats—disparate data sets collected from multiple research projects can be directly compared.

## Abbreviations

DOI: digital object identifier
EMBO: European Molecular Biology Organization
ENCODE: Encyclopedia of DNA Elements
FAIR: findable, accessible, interoperable, and reproducible
FAIR-TLC: findable, accessible, interoperable, reproducible, traceable, appropriately licensed, and interconnected
GA4GH: Global Alliance for Genomics and Health
GEO: Gene Expression Omnibus
IMEx: International Molecular Exchange
ISB: International Society for Biocuration
NCBI: National Center for Biotechnology Information
ORCID: Open Researcher and Contributor ID
PDF: portable document format
UMLS: Unified Medical Language System
wwPDB: Worldwide Protein Database
